# The situation of older Emergency Department patients. Results from a participatory observation study

**DOI:** 10.1093/eurpub/ckac130.110

**Published:** 2022-10-25

**Authors:** J Deutschbein, A Wagenknecht, A Schneider, M Moeckel, L Schenk

**Affiliations:** 1 Institute of Medical Sociology and Rehabilitation Science, Charité - Universitätsmedizin Berlin, Berlin, Germany; 2 Departments of Emergency Medicine, Charité - Universitätsmedizin Berlin, Berlin, Germany

## Abstract

**Background:**

Elderly patients make up a substantial share of Emergency Department (ED) populations which will increase steadily in the coming decades. This poses a challenge for EDs that are not designed to care for multimorbid, frail, and care-dependent older patients. However, too little is known about the current situation of older ED patients and their specific needs. This study seeks to explore ED stays of older and geriatric patients from a patient-centered perspective.

**Methods:**

Participatory observations of older patients’ ED stays were conducted in five different EDs in a central district of Berlin. This included the passive company of ED patients aged 65 years and older, as possible from admission to discharge or referral. The sampling strategy followed the logic of theoretical sampling. Observation notes were captured in a semi-structured protocol and subjected to systematic, comparative analysis based on the Grounded Theory approach.

**Results:**

N = 71 cases of older ED patients were included. Patients’ mean age was 80 years and 52% were female. The total observation time amounted to 332 hours, the mean observation time was 4 hours and 40 minutes. Long waiting hours and uncertainty about the further course turned out to be burdensome for the patients. Other problems were the dependency of patients in their ability to satisfy basic needs such as toileting and hydration. Personnel mostly tried to address these needs but did not always have the capacities.

**Conclusions:**

Like most health care institutions, EDs need to prepare for the consequences of aging societies. Older patients are more vulnerable to stressful situations such as ED stays and depend on more attention and nursing support. ED staff often lack the resources for this. Strategies are needed to adjust ED structures and processes to the specific needs of older patients. This includes the prevention of ED stays.

**Key messages:**

- ED stays can be particularly burdensome for elderly patients. There is a need for strategies to develop patient-centered and age-sensitive ED structures.
- Participatory observation is a productive method for patient-centered health research. Its methodological potential and challenges should be further analyzed and discussed.

